# Real-world Lyme disease testing results using modified vs standard two-tier test protocols

**DOI:** 10.1371/journal.pone.0327376

**Published:** 2025-06-30

**Authors:** Yonghong Li, Zhen Chen, Carmen H. Tong, Ky Van, Robert S. Jones, Lance A. Bare

**Affiliations:** Quest Diagnostics, San Juan Capistrano, California, United States of America; University of Toledo College of Medicine and Life Sciences, UNITED STATES OF AMERICA

## Abstract

The modified two-tier test (MTTT) and standard two-tier test (STTT) protocols are being used for Lyme disease serology testing in the clinic. We aimed to compare the real-world testing results of MTTT, a recently approved protocol, vs STTT, a mainstay protocol over the past decades. To this end, we obtained results of Lyme disease testing performed in a US national reference laboratory in 2022 and 2023 and constructed a matched cohort with 66,708 individuals tested using MTTT and 66,708 individuals tested using STTT. We found that, compared with STTT, MTTT identified more test positives in adults aged 18 and older and similar number of test positives in the children and adolescents. The odds ratio (95% confidence interval) of testing positive using MTTT vs STTT was 1.88 (1.79–1.98) in adults and 1.09 (0.97–1.23) in non-adults. In addition, more patients tested positive for immunoglobulin M antibody alone or positive for both immunoglobulin M and immunoglobulin G antibodies using MTTT than STTT.

## Introduction

Lyme disease, the most common tick-borne disease in the United States, is caused by the infection of *Borrelia burgdorferi* sensu lato from the bites of the infected ticks [1]. In 2023, more than 89,000 cases were reported to the Centers for Disease Control and Prevention (CDC), and during 2010–2018, approximately 476,000 Lyme disease patients might have been diagnosed and/or treated each year according to an analysis of insurance claims data [2]. In patients with a potential recent exposure in the endemic regions such as the Northeast, Upper Midwest, and Mid-Atlantic states [3], Lyme disease may be readily diagnosed by the presence of a distinctive erythema migrans rash in the early stage of infection. However, as recommended by the Infectious Diseases Society of America, American Academy of Neurology, and American College of Rheumatology [4], serological testing for the antibodies to the bacteria is useful for the diagnosis of individuals without the typical skin rash or in later disease stage.

Serological testing can be done by the standard two-tier test (STTT) protocol and the modified two-tier test (MTTT) protocol [5]. STTT uses an enzyme immunoassay (EIA) in the first step to screen the total antibody and the Western blot in the second step to detect immunoglobulin (Ig)M and IgG antibodies, whereas MTTT uses different EIAs in the first and second steps. Although it has been the mainstay protocol over the past decades, STTT has significant limitations: low sensitivity in detecting early-stage disease, technical complexity (resulting in long turnaround time) and subjective Western blot interpretation (leading to incorrect results) [6]. Compared with STTT, MTTT provides greater sensitivity with similar specificity in detecting early infection [7–12].

Since the clearance by the US Food and Drug Administration (FDA) of tests using the MTTT protocol and recommendation by the CDC in 2019 [13], MTTT has been increasingly adopted in routine clinical testing [14]. Given the greater sensitivity using MTTT, its adoption in routine clinical testing is expected to have significant impact on the diagnosis and surveillance of Lyme disease. Test positivity alone in high-incidence regions is now considered acceptable evidence in the surveillance of Lyme disease [15]. However, it is unknown to what extent MTTT affects Lyme disease test positivity compared with STTT in routine clinical testing in the US. Therefore, we conducted a comparative analysis of test results using MTTT and STTT in a national reference laboratory.

## Methods

### Study design

This study retrospectively analyzed de-identified results of Lyme disease serology testing in a clinical setting. The study protocol was reviewed by the WCG Institutional Review Board, an independent ethical review board (www.wcgclinical.com), and the need for authorization was waived by the IRB as this study was deemed exempt based on federal regulation 45 CFR Parts 46, 160, and 164. Informed consent was not obtained because the data were analyzed anonymously.

### Data source

Test codes for *Borrelia burgdorferi* were used to query the Quest Diagnostics test database to identify individuals who were tested for Lyme disease. Data were accessed for research purposes between March 11, 2024 and September 9, 2024 by authors ZC and KV who had access to information that could identify individual participants during data collection but only extracted and analyzed the minimum information necessary for the study. The test codes included MTTT and STTT corresponding to current serologic testing recommendations for clinicians. The tests used for the MTTT protocol were from Zeus Scientific (Branchburg, NJ, USA) with the first-tier EIA test using *Borrelia* VlsE1/pepC10 IgG/IgM test system and separate IgG and IgM EIAs for the second tier. The screening test used for the STTT protocol was based on the Liaison Total Antibody Plus chemiluminescent immunoassay (DiaSorin, Stillwater, MN, USA), detecting both IgG and IgM antibodies to *Borrelia burgdorferi*. Samples with results ≥0.90 (reference range) were reflexed to *Borrelia burgdorferi* IgG and IgM immunoblots from Gold Standard Diagnostics (Davis, CA, USA). These tests were cleared for use by the FDA. Test positivity was determined according to the CDC guidelines: MTTT positivity required a positive or equivocal test result from both steps. STTT positivity required a positive or equivocal test result from the first step and a positive test result from the second step. In 2022 and 2023, Quest Diagnostics offered both MTTT and STTT and performed serology testing per the request of the ordering physicians.

### Study cohort and analyses

Quest Diagnostics test database was queried for Lyme disease serology testing as previously described [14]. Individuals identified had a test done in the year 2022 or 2023 but did not have a test done in the previous 5 years. If an individual had multiple tests done in the same year, only the first test result was analyzed. Individuals who did not follow the 2-tier protocols were excluded. A matched cohort was then constructed from remaining individuals after 1–1 matching MTTT with STTT by age, sex, state, and test year and month. The matching states included individual high-incidence state Connecticut, Massachusetts, New Jersey, New York, Pennsylvania, other high-incidence states as a group (District of Columbia, Delaware, Maryland, Maine, Minnesota, New Hampshire, Rhode Island, Virginia, Vermont, Wisconsin, and West Virginia), and remaining low-incidence states. Summary statistics were performed in Excel 365 (Microsoft Corporation). Logistic regression, without and with adjustment for baseline variables, was performed using SAS statistical software version 9.4 (SAS Institute, Inc) to assess the odds ratio of testing positive using MTTT vs STTT. Although matching was performed on the individual level, we did not compare test results of the individually matched pairs. Instead, all analyses were performed at the population level for the matched cohort.

## Results

**Fig 1:**
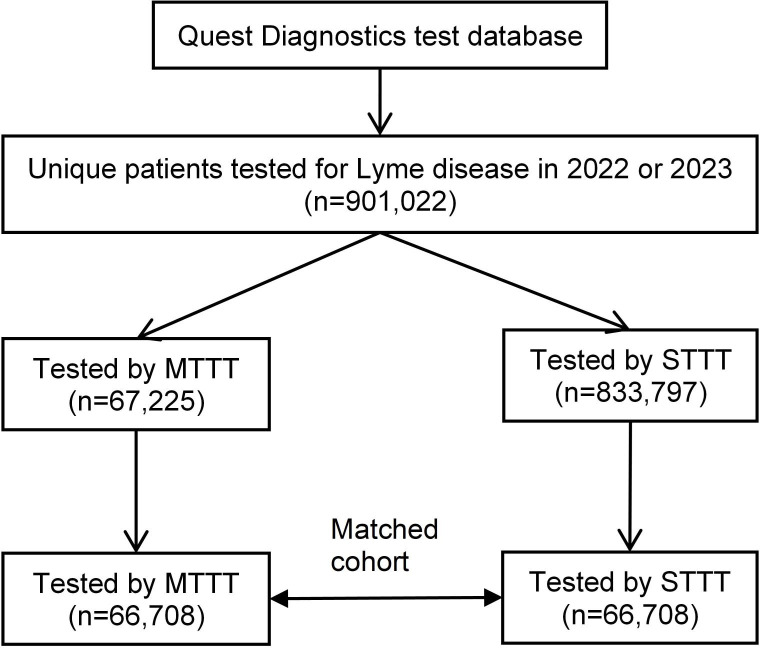
Flow chart of the study cohort. Patients tested using the modified two-tier test (MTTT) protocol and the standard two-tier test (STTT) protocol were matched by age, sex, test month and year, and state of residence.

To compare test results using MTTT and STTT, we constructed a study cohort as illustrated in Fig 1. First, we identified a total of 1,614,880 Lyme disease serology tests done in the years 2022 and 2023. From these tests, we excluded 112,209 repeat tests that were done for the same individuals in the same year, 300,881 individuals who had a test done in the previous 5 years, and 300,768 individuals who did not undergo testing using the 2-tiered protocols. Remaining individuals included 67,225 (7.5%) tested using MTTT and 833,797 (92.5%) tested using STTT. A major difference in the use of these protocols was that out of all individuals tested using MTTT, 40.3% were in Massachusetts; in contrast, out of all individuals tested using STTT, 9.7% were in Massachusetts. However, most of the tested individuals resided in the 5 Northeastern states (Connecticut, Massachusetts, New Jersey, New York and Pennsylvania) for both protocols: 67.0% for MTTT and 64.0% for STTT. Overall, test positivity was 8.1% among those tested using MTTT and 3.7% using STTT. Second, from the 901,022 individuals identified, we constructed a matched cohort consisting of 66,708 individuals tested using MTTT and 66,708 individuals tested using STTT by age, sex, test year and month, and state (grouped by incidence) (Table 1). Overall, the baseline characteristics of the matched cohort are consistent with typical Lyme disease testing pattern [14]; for example, the matched cohort had more females tested, more people tested in summer (June to August), and more people tested in high-incidence states.

**Table 1:** Baseline characteristics of the matched cohort.

Characteristic	Individuals in each test group (n = 66,708), no. (%)
Age, years	
<10	2,762 (4.1)
10-17	3,847 (5.8)
18-29	7,576 (11.4)
30-64	35,857 (53.8)
≥65	16,666 (25.0)
Sex	
Female	37,575 (56.3)
Male	29,133 (43.7)
Year	
2022	26,718 (40.1)
2023	39,990 (59.9)
Month	
Mar-May	13,744 (20.6)
Jun-Aug	26,678 (40.0)
Sept-Nov	16,770 (25.1)
Dec-Feb	9,516 (14.3)
State	
High incidence	51,189 (76.7)
Low incidence	15,519 (23.3)

As expected, for both MTTT and STTT, test positivity rate was higher in the children than other age groups, males than females, summer than other seasons, and states with high than low Lyme disease incidence (Table 2). However, among all individuals tested, positivity rate was higher using MTTT than STTT: 8.0% using MTTT vs 4.8% using STTT. Therefore, MTTT identified 67.6% more test-positive individuals than did STTT. However, test positivity rates using MTTT and STTT were nearly identical or similar in the children/adolescents (Table 2). The odds ratio (95% confidence interval) of testing positive using MTTT vs STTT was 1.88 (1.79–1.98) in adults aged 18 and older and 1.09 (0.97–1.23) in non-adults. For other baseline characteristics, the higher test positivity rate using MTTT than STTT was observed in both males and females, both years 2022 and 2023, all 4 seasons, and both states with high and low Lyme disease incidence (Table 2 and S1 Table).

**Table 2:** Test positivity using MTTT vs STTT.

Characteristic	Test positive, no. (%)		Odds ratio (95%CI) (MTTT vs STTT)
	MTTT	STTT	
Entire cohort	5,368 (8.0)	3,202 (4.8)	1.74 (1.66-1.82)
Age, years			
<10	305 (11.0)	303 (11.0)	1.01 (0.85-1.19)
10-17	337 (8.8)	290 (7.5)	1.18 (1.00-1.39)
18-29	623 (8.2)	320 (4.2)	2.03 (1.77-2.33)
30-64	2,580 (7.2)	1,377 (3.8)	1.94 (1.82-2.08)
≥65	1,523 (9.1)	912 (5.5)	1.74 (1.60-1.89)
Sex			
Female	2,343 (6.2)	1,267 (3.4)	1.91 (1.78-2.04)
Male	3,025 (10.4)	1,935 (6.6)	1.63 (1.53-1.73)
Year			
2022	2,120 (7.9)	1,086 (4.1)	2.03 (1.89-2.19)
2023	3,248 (8.1)	2,116 (5.3)	1.58 (1.50-1.67)
Month			
Mar-May	785 (5.7)	350 (2.5)	2.32 (2.04-2.64)
Jun-Aug	2,949 (11.1)	1,962 (7.4)	1.57 (1.48-1.66)
Sept-Nov	1,094 (6.5)	645 (3.8)	1.75 (1.58-1.93)
Dec-Feb	540 (5.7)	245 (2.6)	2.28 (1.95-2.66)
State			
High incidence	4,777 (9.3)	2,932 (5.7)	1.69 (1.62-1.78)
Low incidence	591 (3.8)	270 (1.7)	2.24 (1.93-2.59)

Abbreviations: CI, confidence interval; MTTT, modified two-tier test; STTT, standard two-tier test.

**Fig 2:**
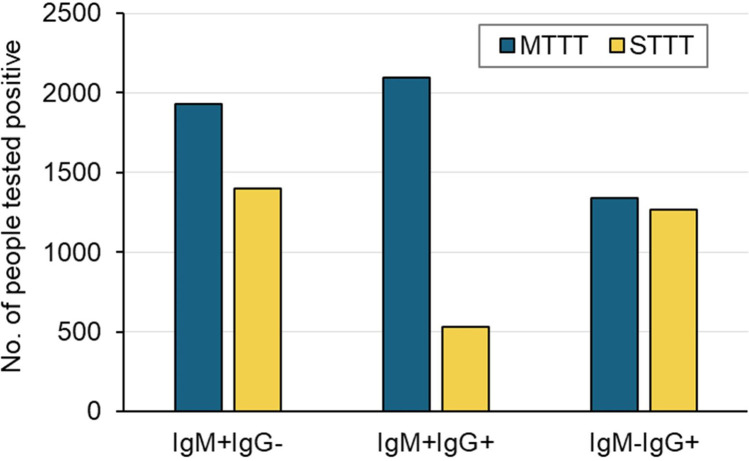
Comparison of IgM and/or IgG positivity using the modified two-tier test (MTTT) protocol with the standard two-tier test (STTT) protocol. Distributions of IgM and/or IgG among individuals who tested positive are shown.

We also compared the number of individuals with specific IgM and/or IgG antibodies detected using MTTT and STTT (Fig 2). More patients tested positive for IgM antibody alone or positive for both IgM and IgG antibodies using MTTT than STTT, whereas the number of patients tested positive for IgG antibody alone was similar. Among individuals who tested positive, the proportion of those who tested positive for IgM (*i.e.*, IgM + IgG- and IgM + IgG+) was higher using MTTT (75.0%) than STTT (60.4%) and the proportion of those who tested positive for IgG alone (*i.e.*, IgM-IgG+) was lower using MTTT (25.0%) than STTT (39.6%). Detection of both IgM and IgG antibodies accounted for 39.0% of the individuals who tested positive using MTTT vs 16.7% of the individuals who tested positive using STTT.

## Discussion

In this retrospective study, we compared the Lyme disease test positivity using MTTT vs STTT in patients referred for routine clinical testing in a large national reference laboratory. We found that more patients tested positive for IgM antibody alone or positive for both IgM and IgG antibodies using MTTT than STTT. This observation is consistent with the reports that MTTT is more sensitive in detecting early-stage infection [7–12]. In addition, we found that MTTT positivity rate was 67.6% higher than STTT positivity rate among all individuals tested. However, positivity rates were not significantly different among non-adults, which is consistent with a previous report [16]. These observations suggest that the number of reported Lyme disease cases will be significantly increased when MTTT is more broadly adopted.

Since the interpretation for MTTT is more permissive in the confirmation test than that for STTT, higher test positivity from MTTT may be expected. The higher MTTT test positivity observed in this study is also consistent with previous reports. For example, in a study of 2,932 samples submitted for clinical testing at 3 US laboratories, 9.7% tested positive using an MTTT protocol vs 6.1% using the STTT protocol [9], translating to 58.1% more test-positives using MTTT. In another study of 2,196 samples submitted for clinical testing in Canada, MTTT detected 28% more cases of early infection [7]. The difference in positivity ratios in these studies could be attributed, in part, to differences in the specific assays used (*i.e.*, differences in their sensitivity and specificity in detecting Lyme disease). Although these 3 studies all used MTTTs from Zeus Scientific, the US-based studies used individual IgM and IgG EIAs in the second step whereas the Canadian study used total IgM/IgG EIA in the second step. The STTTs used in these studies also differed in their sources. The difference in positivity ratios in these studies could also be attributed, in part, to differences in the characteristics of the individuals tested such as age.

This study has some limitations. First, we did not have access to clinical information such as the onset of Lyme disease symptoms that may help interpret the serology test results. We could not rule out false positive test results for individuals who did not have Lyme disease. We also could not rule out false negative test results for individuals who did have Lyme disease. Second, we indirectly compared results from different groups of individuals who were tested using either MTTT or STTT. An ideal study would be a head-to-head comparison of the two test protocols. Such a study has been reported previously [9] and is out of scope of this study designed to assess the impact of adoption of MTTT in real-world testing. A direct comparison in a cohort as large as this study is also infeasible. Third, selection of test protocols might have been influenced by unknown factors such as patient care setting, healthcare provider preference and insurance coverage. Fourth, we compared a specific MTTT from Zeus Scientific with STTT; consequently, the results may not be extended to MTTTs from other manufacturers [17]. A recent study showed that MTTT from Diasorin was less sensitive than MTTT from Zeus Scientific [18]. Therefore, the positivity ratio between Diasorin MTTT and STTT might be smaller than observed in this study.

However, this study also has several strengths. First, the sample size in this study is large (larger than any reported study in peer-reviewed literature comparing performance of MTTT and STTT), and the test results are from a single test provider using the same assays (which could help reduce inter-laboratory variation). Consequently, the test positivity estimates from this study are likely to be reliable. Second, this study included patients in routine clinical testing from all geographic regions and therefore the results may be extended to the overall US population. Third, the individuals included in this study had not been tested for Lyme disease in the prior 5 years at Quest Diagnostics (although some might have had a test done in other laboratories); therefore, the test-positives observed in this study might better represent incident cases than in studies that included repeat testing from the same individuals.

In conclusion, in this retrospective analysis of the test results of patient specimens submitted for routine clinical testing in the US, Lyme disease test positivity in adult patients is much higher using the MTTT protocol than the STTT protocol.

## Supporting information

S1 Table Adjusted odds ratios of testing positive using MTTT vs STTT. (DOCX)

S2 Dataset Matched cohort.
(XLSX)
