# Supplementary material for: Real-world Lyme disease testing results using modified vs standard two-tier test protocols
Source: PLoS One. 2025 Jun 30;20(6):e0327376. doi: 10.1371/journal.pone.0327376 (PMC12208425; doi:10.1371/journal.pone.0327376)
Supplement: S1 Table — (DOCX) [file pone.0327376.s001.docx]

**S1 Table.** Adjusted odds ratios of testing positive using MTTT vs STTT.

| **Characteristic** | **Adjusted odds ratio (95% CI): MTTT vs STTT** |
| --- | --- |
| Entire cohort | 1.76 (1.68-1.84) |
| Age, years |  |
| <10 | 1.01 (0.85-1.19) |
| 10-17 | 1.18 (1.00-1.40) |
| 18-29 | 2.05 (1.79-2.36) |
| 30-64 | 1.98 (1.85-2.11) |
| ≥65 | 1.76 (1.61-1.91) |
| Sex |  |
| Female | 1.93 (1.79-2.06) |
| Male | 1.65 (1.55-1.75) |
| Year |  |
| 2022 | 2.06 (1.91-2.23) |
| 2023 | 1.60 (1.51-1.70) |
| Month |  |
| Mar-May | 2.33 (2.05-2.65) |
| Jun-Aug | 1.58 (1.49-1.68) |
| Sept-Nov | 1.76 (1.59-1.95) |
| Dec-Feb | 2.30 (1.97-2.68) |
| State |  |
| High incidence | 1.71 (1.63-1.80) |
| Low incidence | 2.25 (1.94-2.60) |

Abbreviations: CI, confidence interval; MTTT, modified two-tier test; STTT, standard two-tier test.
